# Clinical and Laboratory Development of Echinocandin Resistance in *Candida glabrata*: Molecular Characterization

**DOI:** 10.3389/fmicb.2019.01585

**Published:** 2019-07-11

**Authors:** Olga Rivero-Menendez, Patricia Navarro-Rodriguez, Leticia Bernal-Martinez, Gema Martin-Cano, Laura Lopez-Perez, Isabel Sanchez-Romero, Ana Perez-Ayala, Javier Capilla, Oscar Zaragoza, Ana Alastruey-Izquierdo

**Affiliations:** ^1^Mycology Reference Laboratory, National Centre for Microbiology, Instituto de Salud Carlos III, Madrid, Spain; ^2^Unitat de Microbiologia, Facultat de Medicina i Ciències de la Salut, Universitat Rovira i Virgili, Tarragona, Spain; ^3^Spanish Network for the Research in Infectious Diseases (RD16CIII/0004/0003), Instituto de Salud Carlos III, Madrid, Spain; ^4^Hospital Universitario Puerta de Hierro, Madrid, Spain; ^5^Hospital Universitario 12 de Octubre, Madrid, Spain

**Keywords:** *Candida glabrata*, echinocandins, antifungal resistance, *FKS*, *MSH2*, genotyping, anidulafungin, micafungin

## Abstract

The pathogenic yeast *Candida glabrata* has become a public health issue due to the increasing number of echinocandin resistant clinical strains reported. In this study, acquisition and development of resistance to this antifungal class were studied in serial *C. glabrata* isolates from five patients admitted in two Spanish hospitals with a resistant profile against echinocandins associated with different mutations in hot-spot 1 of *FKS2* gene. For two of these patients susceptible *FKS* wild-type isolates obtained prior to resistant ones were also investigated. Isolates were genotyped using multilocus sequence typing and microsatellite length polymorphism techniques, which yielded comparable results. Susceptible and resistant isolates from the same patient had the same genotype, being sequence type (ST) 3 the most prevalent among them. Isolates with different *FKS* mutations but the same ST were present in the same patient. *MSH2* gene alterations were also studied to investigate their correlation with antifungal resistance acquisition but no association was found with antifungal resistance nor with specific genotypes. *In vitro* exposure to increasing concentrations of micafungin to susceptible isolates developed colonies carrying *FKS* mutations in agar plates containing a minimum concentration of 0.06 mg/L of micafungin after less than 48 h of exposure. We investigated the correlation between development of resistance and genotype in a set of susceptible strains after being *in vitro* exposed to micafungin and anidulafungin but no correlation was found. Mutant prevention concentration values and spontaneous growth frequencies after selection with both echinocandins were statistically similar, although *FKS* mutant colonies were more abundant after micafungin exposure (*p* < 0.001). Mutation S663P and F659 deletion were the most common ones found after selection with both echinocandins.

## Introduction

Infections caused by *Candida* species, extensively referred to as candidiasis, have been described as the most common fungal disease globally (Pappas et al., 2018). Although *Candida albicans* is the species causing the highest number of infections in clinical settings, an increasing prevalence of other *Candida* species has been reported in the last years, being *Candida glabrata* the second most common species isolated from invasive candidiasis in North America and central and northern countries in Europe (Pfaller et al., 2012; Asmundsdottir et al., 2013; Lortholary et al., 2014; Milazzo et al., 2014; Castanheira et al., 2016). In Spain, only *C. albicans* and *Candida parapsilosis* are more frequently isolated than *C. glabrata* from patients with fungemia (Guinea et al., 2014).

Echinocandins are the first line antifungal therapy against *C. glabrata* infections, as this species generally presents low susceptibility to azole drugs. Echinocandins non-competitively inhibit the 1–3-β-D-glucan synthase, which is responsible for the synthesis of β-glucan polymers that confer integrity to the fungal cell wall. Nevertheless, an ever-growing number of echinocandin resistant clinical isolates have been reported worldwide in the last years and population studies in the United States and Denmark have shown an increase in echinocandin resistance rate (Alexander et al., 2013; Farmakiotis et al., 2014; Vallabhaneni et al., 2015; Astvad et al., 2017), which is conferred by the presence of point mutations in specific regions (denominated as hot-spots) of *FKS* genes, which encode this enzyme’s catalytic subunits (Katiyar et al., 2012).

*FKS* mutations have been reported to correlate with elevated *in vitro* minimal inhibitory concentrations (MICs) and clinical failure (Alexander et al., 2013), yet an explanation for this increase in echinocandin resistant strains has not been proved. Several possibilities are being studied, such as strains proneness to acquire resistance as an answer to echinocandin exposure (Bordallo-Cardona et al., 2017, 2018a,c; Shields et al., 2019), the existence of hidden reservoirs in the human body of echinocandin resistant *C. glabrata* isolates (Shields et al., 2014; Grau et al., 2015; Jensen et al., 2015; Healey et al., 2017) or molecular mechanisms like *MSH2* mutator phenotype (Delliere et al., 2016; Healey et al., 2016b; Byun et al., 2018; Hou et al., 2018; Singh et al., 2018; Bordallo-Cardona et al., 2019).

Multilocus sequence typing (MLST) and microsatellite length polymorphism (MLP) have been described as typing methodologies with high discrimination power (Dodgson et al., 2003; Foulet et al., 2005; Abbes et al., 2012) for assesing *C. glabrata* strain relatedness.

The objective of the present study was to investigate the antifungal susceptibility, molecular mechanisms of echinocandin resistance and strain relatedness of a series of *C. glabrata* sequentially isolated from patients admitted in two hospitals in Madrid; and also the potential development of echinocandin resistance of susceptible *C. glabrata* isolates collected from 2013 to 2017 after *in vitro* exposure to a range of micafungin and anidulafungin concentrations.

## Materials and Methods

### Yeast Isolates: Patients and Identification

Eighteen *C. glabrata* strains sequentially isolated from five patients admitted in two centers (Hospital Universitario Puerta de Hierro and Hospital Universitario 12 de Octubre, both located in Madrid, Spain) were selected for showing a resistance profile against echinocandins. For two of these patients previous susceptible isolates were also available and analyzed. 89% (16/18) of them were obtained from blood cultures, while the two remaining were isolated from a catheter (one isolate from Patient 1) and from ascitic liquid (one isolate from Patient 2) (Table 1). For *in vitro* exposure to micafungin and anidulafungin assays, 14 *C. glabrata* strains collected from 2012 to 2017 from Hospital Universitario Puerta de Hierro, all isolated from blood cultures except one from ascitic liquid, were chosen for being susceptible to echinocandin drugs. All strains were isolated during routine diagnostic procedures at the hospitals and received at the Mycology Reference Laboratory of the Spanish National Centre for Microbiology. Isolates were characterized by morphological features and confirmed as *C. glabrata* by amplification and sequencing of their ITS1-5.8S-ITS2 regions (White et al., 1990). According to the Law 14/2007 of 3rd July on Biomedical Research and the Recommendation CM/Rec(2016)6 of the Committee of Ministers to member States on research on biological materials of human origin, no informed consent was required as no work was performed neither with samples of human origin nor with clinical data. The Mycology Reference Laboratory directly received fungal strains, isolated from the patients as routine diagnostic procedures in the hospital and referred to the National Centre for Microbiology according to routine procedures.

**Table 1:** *Candida glabrata* sequential isolates from five patients admitted in two hospitals in Madrid: isolation dates, anatomic sources, *FKS2* alterations, *in vitro* susceptibility to echinocandins and fluconazole performed by EUCAST and genotyping results by MLST and MLP.

Hospital	Patient	Strain	Isolation date	Anatomic source	*FKS 2* alteration	MIC EUCAST (mg/L)				Typing	
						ANF	CPF	MCF	FLC	MLP^∗^ (bp)	MLST (ST)
Hospital	1	CNM-CL9829	14/03/16	Blood culture	–	0.007	0.25	0.007	4	205-243-134-267-	ST3
Universitario Puerta		CNM-CL9835	17/03/16	Catheter	–	0.015	0.25	0.007	4	262-325	
de Hierro, Madrid,		CNM-CL9975	17/04/16	Blood culture	D666H	**0.125**	0.5	0.03	4		
Spain		CNM-CL9877	17/06/16	Blood culture	L664R	**0.125**	1	**0.06**	2		
		CNM-CL9889	07/07/16	Blood culture	L664R	**0.125**	1	**0.125**	2		
	2	CNM-CL9857	21/05/16	Blood culture	–	0.03	0.25	0.007	2	205-243-134-267-	ST3
		CNM-CL9883	23/06/16	Ascitic fluid	D666E	**0.25**	1	**0.06**	4	262-325	
		CNM-CL9897	17/07/16	Blood culture	S663P	**2**	>16	**>2**	32		
	3	CNM-CL9931	21/10/16	Blood culture	ΔF659	**2**	>16	**2**	2	187-251-122-270-	ST2
		CNM-CL9939	14/11/16	Blood culture	ΔF659	**2**	>16	**2**	**64**	265-296	
		CNM-CL9991	23/11/16	Blood culture	ΔF659	**2**	>16	**2**	**>64**		
	4	CNM-CL9932	21/10/16	Blood culture	S663P	**2**	>16	**2**	32	205-243-134-267-	ST3
		CNM-CL9981	12/11/16	Blood culture	S663P	**2**	>16	**>2**	32	262-325	
		CNM-CL9992	24/11/16	Blood culture	S663P	**2**	>16	**>2**	**64**		
Hospital	5	CNM-CL9646	25/02/15	Blood culture	D666N	**0.125**	0.5	**0.06**	**64**	237-236-128-270-	ST149
Universitario 12 de		CNM-CL9775	11/11/15	Blood culture	D666N	**0.125**	0.5	**0.06**	2	262-290	
Octubre, Madrid,		CNM-CL9988	16/11/16	Blood culture	D666N	**0.25**	2	**0.06**	2		
Spain		CNM-CL10047	16/01/17	Blood culture	ΔF659 + D666N	**>4**	16	**2**	**64**		

*CNM-CL, Yeast Collection of the Spanish National Center for Microbiology; ANF, anidulafungin; CPF, caspofungin; MCF, micafungin; FLC, fluconazole; MLP, Microsatellite Length Polymorphism; bp, base pairs; MLST, Multilocus Sequence Typing; ST, sequence type. In bold letters, MIC values that are above the EUCAST clinical breakpoints stablished for those antifungals. ^∗^MLP: ERG3-MTI-RPM2-GLM4-GLM5-GLM6*.

### Antifungal Susceptibility Testing

Minimal inhibitory concentrations were determined and confirmed following EUCAST 7.3.1 reference method for yeasts^1^. Antifungals tested were anidulafungin (range 0.007–4 mg/L; Pfizer, Madrid, Spain), micafungin (range 0.004–2 mg/L; Astellas Pharma Inc., Tokyo, Japan), caspofungin (range 0.032–16 mg/L; Merck Sharp & Dohme, United Kingdom) and fluconazole (range 0.125–64 mg/L; Pfizer, Madrid, Spain).

*Candida krusei* ATCC 6258 and *C. parapsilosis* ATCC 22019 were used as quality control strains in all test performed. The optical density of the inoculated plates was determined after 24 and 48 h of incubation at 35°C in a humid atmosphere, and strains were classified as susceptible or resistant according to clinical breakpoints established by EUCAST for *C. glabrata*: MIC > 0.032 mg/L for micafungin, MIC > 0.064 mg/L for anidulafungin and MIC > 32 mg/L for fluconazole^2^.

### DNA Extraction and *FKS* Sequencing

Genomic DNA of all isolates was extracted using the phenol-chloroform method (Tang et al., 1992). Molecular mechanisms of echinocandin resistance were studied by amplifying hot-spot regions 1 and 2 of *FKS1* and *FKS2* (Thompson et al., 2008; Zimbeck et al., 2010; Duran-Valle et al., 2012; Bizerra et al., 2013) as previously described with the following modifications: PCR reaction mixtures contained 25 ng of DNA, 0.2 μM of each primer, 0.2 μM of deoxynucleoside triphosphate (Roche, Spain), 5 μL of PCR 10× buffer (Applied Biosystems, Foster City, CA, United States), 2 mM of MgCl_2_ (Applied Biosystems, Foster City, CA, United States), 5.2% DMSO and 2.5 U of Taq DNA polymerase (Applied Biosystems, Foster City, CA, United States) in a final volume of 50 μL. PCRs conditions used were set as previously described (Duran-Valle et al., 2012), with an annealing temperature of 52°C for hot-spot regions 1 and 2 of *FKS1*, 53°C for hot-spot region 1 of *FKS2* and 58°C for hot-spot region 2 of *FKS2*. PCR amplicons were purified using Illustra ExoProStar 1-step (GE Healthcare Life Science, United Kingdom), and were sequenced after by Sanger method with an ABI3730XLsequencer (Applied Biosystems, Foster City, CA, United States). DNA sequences were analyzed with DNAStar Lasergene 12 software (DNAStar Inc., United States), and queried against *FKS1* (GenBank number CAGL0G01034g) and *FKS2* (GenBank number CAGL0K04037g) sequences of the type strain CBS 138^3^.

### Assessment of Pooled Reservoir of Mixed Resistant Isolates

For two patients with isolates harboring two different *FKS* mutations, the possible coexistence of diverse populations within the same sample was studied by randomly isolating ten colonies from the original samples sent from the hospital for DNA extraction and *FKS* amplification and sequencing.

### Genotyping by MLST

Six housekeeping gene loci (*FKS, LEU2, NMT1, TRP1, UGP1*, and *URA3*) were studied for all isolates as previously described (Dodgson et al., 2003), with the following modifications: PCR reaction mixtures contained 25 ng of DNA, 1 μM of each primer, 0.05 μM of deoxynucleoside triphosphate, 5 μL of PCR 10× buffer, 2 mM of MgCl_2_, and 2.5 U of Taq DNA polymerase in a final volume of 50 μL. 5.2% of DMSO was added only to amplify *NMT1*. PCR conditions were set as described, but with an annealing temperature of 62°C for *FKS* and *URA3*. DNA sequences obtained were compared to *C. glabrata* MLST database^4^ to assign an allele number for each locus in order to define a sequence type (ST) or genotype according to the isolates’ allelic profile.

### Genotyping by MLP

Six short tandem repeat markers described for *C. glabrata* (*ERG3, MTI, RPM2, GLM4, GLM5*, and *GLM6*) (Foulet et al., 2005; Abbes et al., 2012) were amplified by PCR for all isolates using forward labeled primers as previously described (Foulet et al., 2005; Abbes et al., 2012; Duran-Valle et al., 2012) with the following modifications: *GLM5* was labeled with HEX and *GLM6* with NED fluorochromes. PCR reaction mixtures contained 20 ng of DNA, 0.2 μM of deoxynucleoside triphosphate, 2 μL of PCR 10× buffer, 2.5 mM of MgCl_2_, 5 U of Taq DNA, 0.25 μM of *RPM2* and *ERG3* primers and 1 μM of *MTI, GLM4, GLM5*, and *GLM6* primers in a final volume of 20 μL. PCR program used from amplifying all markers consisted on an initial step of 5 min at 95°C, followed by 40 cycles of 95°C for 30 s, 57°C for 1 min and 72°C for 1 min, and an additional step of 7 min at 72°C. Amplicons were sized by capillary electrophoresis using Hi-Di formamide (Applied Biosystems, Foster City, CA, United States) and ROX 500 (Applied Biosystems, Foster City, CA, United States) as internal size standard, as described (Duran-Valle et al., 2012). Reactions were analyzed in duplicate, and fragment sizes were calculated using Peak Scanner software 1.0 (Applied Biosystems, Foster City, CA, United States).

### *MSH2* Sequencing

*MSH2* gene of sequentially isolated strains from patients was amplified and sequenced as previously described (Healey et al., 2016b), and DNA sequences were queried against *MSH2* (Genbank number CAGL0I07733g) sequence of CBS 138 type strain.

### *In vitro* Exposure to Growing Concentrations of Micafungin and Anidulafungin and Analysis of Generated Isolates

The potential development of micafungin and anidulafungin resistance of susceptible *C. glabrata* isolates was studied following a previously reported procedure (Bordallo-Cardona et al., 2017) with some modifications.

Adjusted inocula (2 × 10^9^ to 4 × 10^9^ CFU/mL) from overnight cultures in 7 mL of yeast extract-peptone-dextrose broth of these isolates were cultured on Sabouraud plates containing eight different echinocandin concentrations (from 0.015 to 2 mg/L). Two different sets of experiments were tested at first. Plates at all concentrations were stroked at once with 100 μL of inocula and checked for growth daily for up to 5 days at 35°C. For progressive exposure, the lowest concentration plate was inoculated and checked for growth after 24 h at 35°C. If isolates were observed, they were cultured on a plate containing the next twofold concentration. The procedure was repeated up to highest concentration available.

After exposure, mutant prevention concentration (MPC) and mutant selection window (MSW) were calculated for each isolate as previously reported (Zhao and Drlica, 2001; Drlica, 2003; Bordallo-Cardona et al., 2018c). Briefly, MPCs were defined as the lowest concentration that can totally inhibit fungal growth for each isolate after 5 days of incubation; for calculation purposes, MPC values that exceeded the highest concentration tested were transformed to the next dilution (i.e., if MPC >2 mg/L, it was changed to MPC = 4 mg/L). MSWs were defined as the range of concentrations between the MIC, obtained by EUCAST method, and the MPC for each isolate.

Spontaneous growth frequency was also calculated as the ratio of viable colonies growing on 2 mg/L echinocandin-containing plates and the initial inoculum stroked in them, as some plates containing lower concentrations did not allow the counting of individual colonies.

Micafungin and anidulafungin susceptibility of up to four isolates selected from each growing concentration was performed, and the hot-spot regions 1 and 2 of *FKS1* and *FKS2* genes were sequenced.

### Statistical Analysis

All data obtained after *in vitro* exposure to echinocandins assays were compared using the Wilcoxon signed-rank test and the Fisher’s exact test (IBM SPSS Statistics for Windows, version 22.0; United States), considering as statistically significant a *P-*value of <0.05.

## Results

### *In vitro* Susceptibility and Determination of *FKS* Mutations of Sequentially Isolated Strains

Control strains were within the accepted ranges according to EUCAST QC ranges for all antifungals tested.

As shown in Table 1, a wide range of fluconazole MIC values was found among isolates tested. All strains isolated from Patients 3, 4, and 5 were echinocandin resistant, according to EUCAST breakpoints established for anidulafungin and micafungin. For Patients 1 and 2, echinocandin susceptible isolates were also available and analyzed. The first resistant isolate from Patient 1 was only resistant to anidulafungin.

All resistant isolates harbored an echinocandin resistance related mutation at hot-spot region 1 of *FKS2* gene. Mutations found were S663P (*n* = 4), D666N (*n* = 4), ΔF659 (*n* = 4), L664R (*n* = 2), D666E (*n* = 1), and D666H (*n* = 1). No mutations were found at *FKS1* nor at hot-spot region 2 of *FKS2*. Each mutation was related to a different echinocandin resistant profile, as S663P and ΔF659 showed higher MIC values than the rest of the isolates that harbored other mutations.

### Isolates With Different *FKS* Mutations Can Be Present in the Same Patient

Resistant isolates with different *FKS2* mutations were found in the same patient in two cases (Patient 1: D666H and L664R; and Patient 2: D666E and S663P). *FKS2* gene sequencing of 10 randomly selected colonies from the original samples of those isolates sent from the hospital led to the same *FKS* mutation in all of them, so the absence of a mixed culture of resistant isolates was confirmed.

### MLST and MLP Analysis

Three different STs were differentiated by MLST among the 18 isolates studied (Table 1). All isolates from the same patient had the same genotype. ST3 was found in three out of four patients of one hospital. The other two STs found were ST2 and a recently described ST149.

MLP methodology yield comparable results to those of MLST (Table 1).

### *MSH2* Gene Sequencing

All isolates from the same patient harbored the same SNPs in *MSH2* gene. Non-synonymous loss-of-function combined mutations V239L/A942T were found in all isolates coming from one patient, while the rest of isolates did not harbor any non-synonymous mutations in this gene.

### Behavior of Echinocandin Susceptible *C. glabrata* Isolates From Patients 1 and 2 When *in vitro* Exposed to Growing Concentrations of Micafungin

**FIGURE 1:**
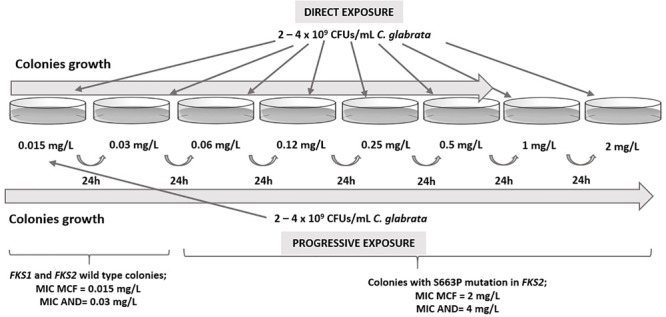
Results from *in vitro* direct and progressive exposure to micafungin of susceptible *C. glabrata* isolates from Patients 1 and 2.

*In vitro* exposure of echinocandin susceptible isolates CNM-CL9829, CNM-CL9835, and CNM-CL9857 from Patients 1 and 2 to micafungin generated *FKS* mutations that conferred echinocandin resistance after less than 48 h of incubation. Progressive exposure allowed the collection of isolates up to 2 mg/L, while colonies in direct exposure grew only up to 0.5 mg/L. All colonies obtained in plates containing 0.015 and 0.03 μg/mL were susceptible to micafungin and anidulafungin and had no mutations in hot-spot regions of *FKS* genes. All isolates growing from 0.06 μg/mL were resistant to both echinocandins and harbored the resistant related mutation S663P in *FKS2* gene (Figure 1).

### Correlation Between Potential Development of Echinocandin Resistance After *in vitro* Exposure to Micafungin and Anidulafungin of Echinocandin Susceptible Isolates of *C. glabrata* and Their Genotype

Table 2 shows genotyping results by MLST and MLP, echinocandin susceptibility by EUCAST, MPC and MSW after *in vitro* direct exposure to micafungin and anidulafungin of fourteen echinocandin susceptible *C. glabrata* strains collected from 2012 to 2017 from Hospital Universitario Puerta de Hierro, total number of colonies analyzed per isolate (up to 4 colonies per concentration) after 5 days of incubation and *FKS* mutations found.

**Table 2:** Isolation year, micafungin and anidulafungin MIC values, mutant prevention concentration (MPC), mutant selection window (MSW), total number of colonies analyzed and *FKS* alterations found in them after 5 days of incubation for each isolate, and their genotype by MLP and MLST.

Strain		Isolation year	Anidulafungin exposure					Micafungin exposure					MLP^∗^ (bp)	MLST (ST)
			MIC (mg/L)	MPC (mg/L)	MSW (mg/L)	Total no. isolates analyzed	*FKS* alteration	MIC (mg/L)	MPC (mg/L)	MSW (mg/L)	Total no. isolates analyzed	*FKS* alteration		
**CNM-CL9210**		2012	0.03	2	0.03–2	14	–	0.007	4	0.007–4	27	2-S663P	237-236-128-270-262-290	149
**CNM-CL9215**		2012	0.03	2	0.03–2	13	–	0.007	4	0.007–4	26	2-S663P	237-236-128-270-262-290	149
**CNM-CL9269**		2012	0.03	4	0.03–4	26	2-S663P	0.007	4	0.007–4	27	2-S663P	205-243-134-267-262-325	3
**CNM-CL9332**		2013	0.03	4	0.03–4	19	1-S629P; 2-F659Y	0.015	4	0.015–4	27	2-S663P	215-242-134-282-265-298	19
**CNM-CL9342**		2013	0.06	2	0.06–2	11	–	0.015	0.25	0.015–0.25	13	2-ΔF659	215-242-134-282-265-298	19
**CNM-CL9392**		2013	0.06	4	0.06–4	26	2-S663P	0.015	4	0.015–4	27	2-S663P	215-242-134-282-265-298	19
**CNM-CL9555**		2014	0.03	4	0.03–4	26	2-S663P	0.015	4	0.015–4	20	2-ΔF659	205-243-134-267-262-325	3
**CNM-CL9571**		2014	0.03	2	0.03–2	12	–	0.015	0.5	0.015–0.5	13	–	205-243-134-267-262-325	3
**CNM-CL9780**		2015	0.03	4	0.03–4	25	2-S663P	0.007	4	0.007–4	20	1-S629P; 2-ΔF659; 2-L662W	215-242-134-282-265-298	19
**CNM-CL9785**		2015	0.03	0.5	0.03–0.5	13	–	0.007	4	0.007–4	26	2-S663P	205-243-134-267-262-325	3
**CNM-CL9862**		2016	0.03	0.5	0.03–0.5	10	–	0.007	0.5	0.007–0.5	11	–	230-243-128-270-262-325	6
**CNM-CL9906**		2016	0.03	4	0.03–4	18	2-S663P	0.007	0.5	0.007–0.5	14	2-ΔF659	205-243-134-267-262-325	3
**CNM-CL10190**		2017	0.03	4	0.03–4	16	2-ΔF659	0.007	0.5	0.007–0.5	19	–	205-243-134-267-262-325	3
**CNM-CL10194**		2017	0.03	4	0.03–4	29	2-S663P	0.007	2	0.007–2	26	2-ΔF659; 2-S663P	205-243-134-267-262-325	3
**Global**	**GM**		0.03	2.44		Total = 258		0.01	1.72		Total = 296			
	**Range**		0.03–0.06	0.5–>2	0.03–>2			0.007–0.015	0.25–>2	0.007–>2				

*GM, geometric mean. FKS alteration: (1) mutation in FKS1 gene; (2) mutation in FKS2 gene. ^∗^MLP: ERG3-MTI-RPM2-GLM4-GLM5-GLM6*.

Four different STs were found among these strains. The most common one was ST3, found in half of the isolates (*n* = 7), followed by ST19 (29%, *n* = 4), ST149 (14%, *n* = 2), and ST6 (7%, *n* = 1).

Mutant prevention concentration values after anidulafungin and micafungin exposure differ widely between strains (Table 2), although no significant differences were found between the geometric mean of MPCs after anidulafungin exposure and after micafungin exposure after 5 days of incubation (2.44 mg/L versus 1.72 mg/L).

Geometric mean of spontaneous growth frequency for micafungin-containing plates had no significant difference with that for anidulafungin-containing plates (8 × 10^-8^ versus 4.1 × 10^-8^; *p* = 0.78), and ranges were very similar for both of them (4.1 × 10^-7^ to 3.2 × 10^-9^ in the presence of micafungin and 3.7 × 10^-7^ to 5.3 × 10^-9^ in the presence of anidulafungin).

**FIGURE 2:**
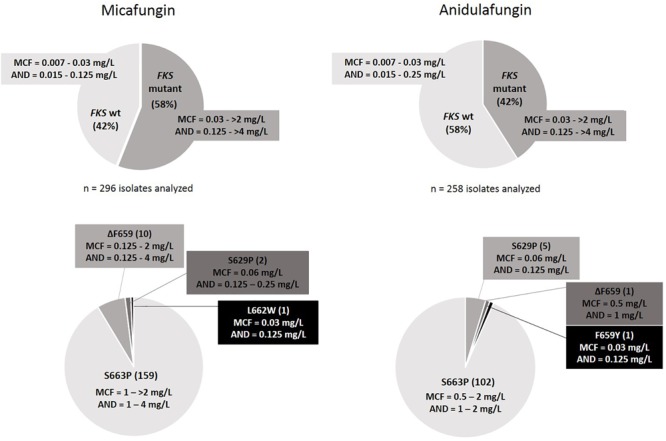
*FKS* mutant and wild-type colony rates with their MIC ranges **(Top)**, and *FKS* mutations found with range of MIC values per mutation **(Bottom)** after *in vitro* exposure to micafungin and anidulafungin.

A total number of 296 and 258 isolates were analyzed after micafungin and anidulafungin exposure, respectively (Figure 2). The lowest concentrations of these antifungals in which resistant colonies harboring *FKS* mutations were found were 0.06 mg/L and 0.12 mg/L, respectively, while they appeared up to the highest concentration tested in both cases, 2 mg/L. 58% of the isolates yielded in micafungin plates harbored *FKS* mutations related to echinocandin resistance, which was a significantly higher number than the 42% of the isolates that did so after anidulafungin exposure (*p* < 0.0001). The most prevalent mutation found was S663P (no significant differences were found among the frequency of occurrence of this substitution after exposure to both echinocandins: *n* = 159 from MCF plates and *n* = 102 from AND plates; 92.4 and 93.5% of *FKS* mutant isolates generated, *p* = 0.8147), followed by ΔF659 (its appearance rate was close to be significantly different after exposure to both antifungals: *n* = 10 from MCF plates and *n* = 1 from AND plates, *p* = 0.0552), F659Y (*n* = 1 from AND plates) and L662W (*n* = 1 from MCF plates) in hot-spot 1 from *FKS2* gene and S629P (*n* = 5 from AND plates and *n* = 2 from MCF plates) in hot-spot 1 from *FKS1* gene. All of these isolates were echinocandin resistant by EUCAST, and their *FKS* mutations and resistance were stable and reproducible after subculturing on antifungal-free plates. The same isolate could develop different *FKS* mutations after exposure. Out of the total number of isolates analyzed after micafungin and anidulafungin exposure, 1 and 19%, respectively, were resistant to micafungin and/or anidulafungin but did not carry any *FKS* mutation.

## Discussion

The increasing number of *C. glabrata* clinical isolates reported showing decreased susceptibility for echinocandins is a growing concern. Recent studies indicate that echinocandin resistance rates among *C. glabrata* clinical isolates have risen worldwide (Kiraz et al., 2010; Bourgeois et al., 2014; Guinea et al., 2014; Orasch et al., 2014; Klotz et al., 2016; Chapman et al., 2017; Hou et al., 2017). Resistance has been reported to easily develop *in vitro* (Bordallo-Cardona et al., 2017, 2018a,c; Shields et al., 2019) and in patients after echinocandin exposure (Dannaoui et al., 2012; Shields et al., 2012; Alexander et al., 2013; Bizerra et al., 2014; Sasso et al., 2017), being conferred by the presence of point mutations in hot-spot regions of *FKS1* and *FKS2* genes (Castanheira et al., 2014; Pham et al., 2014) that have been associated with higher MICs and therapeutic failure (Shields et al., 2012). Our study provides a new insight into the development of echinocandin resistance of *C. glabrata* strains both sequentially isolated from several patients and after *in vitro* exposure to growing concentrations of micafungin and anidulafungin.

### *FKS* Mutations

All isolates from five patients admitted in two Spanish hospitals that were resistant to at least one echinocandin carried mutations in *FKS2* gene (Zimbeck et al., 2010; Castanheira et al., 2014; Locke et al., 2016). The most common ones found were S663P and ΔF659, as in previous studies (Zimbeck et al., 2010; Beyda et al., 2014; Castanheira et al., 2014). Some isolates carried less frequently found substitutions L664R, D666E, and D666N (Castanheira et al., 2014; Prigent et al., 2017; Bordallo-Cardona et al., 2018b) and, although it has been found after *in vitro* exposure to the novel antifungal rezafungin (Locke et al., 2016), we believe that this is the first time that D666H mutation is reported in a clinical isolate.

From two of the studied patients, echinocandin susceptible isolates without mutations in *FKS* genes were detected before the isolation of the resistant ones, indicating that echinocandin resistance could had been acquired due to therapy, as previously reported in other studies (Cleary et al., 2008; Thompson et al., 2008; Ishikawa et al., 2010; Costa-de-Oliveira et al., 2011; Dannaoui et al., 2012; Shields et al., 2012; Alexander et al., 2013; Lewis et al., 2013; Beyda et al., 2014). All isolates with *FKS* mutations had MIC values above the clinical breakpoints established by EUCAST both for anidulafungin and for micafungin, except one isolate that was resistant to anidulafungin but not to micafungin despite harboring an *FKS* mutation (D666H). This case has been described before (Arendrup et al., 2012; Shields et al., 2012; Jensen et al., 2015; Bordallo-Cardona et al., 2017) but should be taken into account when testing only one echinocandin to detect resistance.

Glucan synthase enzyme sensitivity has been described to be affected by *FKS* mutations on different degrees (Garcia-Effron et al., 2009). In this study, S663P and ΔF659 were associated with higher echinocandin MIC values, as previously reported (Arendrup and Perlin, 2014). Isolates harboring D666H, D666N, D666E, and L664R substitutions showed lower MIC values and conferred weaker echinocandin resistance. Patient 5 simultaneously carried ΔF659 and D666N substitutions in CNM-CL10047 isolate, which had high echinocandin MIC values. A double mutation in these two amino acid positions has been previously found (F659S and D666E) (Prigent et al., 2017), but we are reporting the association of ΔF659 and D666N for the first time.

As we found sequential isolates from the same patient carrying different *FKS* mutations, we studied the possible coexistence of a mixed population of resistant isolates within the same sample, confirming its absence. Nevertheless, this experiment had some limitations, as the original samples sent from the hospital could be an already isolated colony. Serial *C. glabrata* isolates from the same patient showing different antifungal resistance profiles due to the selective pressure induced by changes in antifungal treatment have been previously reported (Cho et al., 2015; Imbert et al., 2016).

### Genotyping

Strain relatedness was determined by MLST and MLP, which led to similar results evidencing that both methodologies are equally useful for genotyping purposes, as previously reported (Brisse et al., 2009). Nevertheless, in other investigations a higher number of genotypes were obtained by MLP than by MLST (Hou et al., 2018; Bordallo-Cardona et al., 2019). Isolates from the same patient seemed to have a clonal origin by using these two typing techniques, although the use of next generation sequencing in order to compare their genomes would be necessary to prove if they are isogenic. The most frequent genotype among these patients was ST3, which has been reported as one of the most prevalent STs worldwide (Dodgson et al., 2003; Lott et al., 2012; Hou et al., 2017; Biswas et al., 2018; Byun et al., 2018; Mushi et al., 2018). No association between echinocandin resistance development and genetic type was found, which was in agreement with other reports (Dodgson et al., 2003; Abbes et al., 2012). A recent study has found a link between certain STs and reduced susceptibility to fluconazole (Mushi et al., 2018), something that we did not see when performing fluconazole susceptibility to all the isolates. Genotypes were also independent of the anatomic source of the isolates (Lin et al., 2007).

### MSH2

*MSH2* mismatch repair gene involved in DNA repair has been described as a promoter of the acquisition of resistance to antifungals of *C. glabrata* (Healey et al., 2016b), but in this study all isolates belonging to the same patient had the same *MSH2* gene sequence, regardless their susceptibility pattern to echinocandins. Echinocandin susceptible isolates with and without *MSH*2 mutations yielded echinocandin resistant isolates with *FKS* mutations. Also, *FKS* mutant isolates for three patients had a wild-type *MSH2* gene. Altogether, this supports that echinocandin resistance cannot be explained by *MSH2* mutator phenotype, as previously reported (Delliere et al., 2016; Healey et al., 2016a; Biswas et al., 2018; Byun et al., 2018; Hou et al., 2018; Singh et al., 2018; Bordallo-Cardona et al., 2019). Likewise, no clear association between *MSH2* sequence and increased fluconazole resistance or genotypes was detected on these isolates either (Biswas et al., 2018; Bordallo-Cardona et al., 2019), although a correlation with specific genetic types was previously described (Delliere et al., 2016; Byun et al., 2018; Hou et al., 2018). These results confirm that *MSH2* substitutions may be constitutive variations from the gene rather than resistance-related or genotype-related mutations (Carrete et al., 2019). Still, it cannot be dismissed that *MSH2* may just be one of a higher number of *C. glabrata* genes involved in mismatch repair mechanisms influencing on the development of antifungal resistance, as it happens for other yeasts (Legrand et al., 2007; Boyce et al., 2017).

### *In vitro* Resistance Development

It is of interest to gain a deeper understanding of how *C. glabrata* isolates behave when *in vitro* exposed to echinocandins, and correlate these results with clinical findings or to anticipate to possible clinical cases. Susceptible isolates from Patients 1 and 2 were *in vitro* exposed to a range of growing concentrations of micafungin, obtaining echinocandin resistant and *FKS* mutant colonies after exposure to the lowest micafungin concentration considered resistant by EUCAST (0.06 mg/L) in less than 48 h of incubation. These results are in agreement with those previously reported (Bordallo-Cardona et al., 2017, 2018a). In our study, *FKS* mutations found after *in vitro* micafungin exposure were different from those isolated from the patients, something that evinces *C. glabrata*’s facility to develop resistance and to acquire different mutations under drug pressure.

It has been hypothesized that certain STs may have a better competence than others to acquire resistance through antifungal exposure at different frequencies (Lott et al., 2012; Hou et al., 2017). Therefore, we aimed to compare the potential development of *in vitro* echinocandin resistance of a set of susceptible *C. glabrata* isolates collected in 6 years from one hospital after exposure to a range of micafungin and anidulafungin concentrations, and to correlate this with their genotype. MLST and MLP revealed four different STs among these fourteen isolates. Not all strains isolated on the same year showed the same ST in all cases and no clear trend on the evolution of *C. glabrata* population in this center was found. No statistical differences were found among MPC values and spontaneous growth frequencies for both agents, something in concordance with previous results published (Bordallo-Cardona et al., 2018c). Results in our study varied between strains, being echinocandin resistant colonies harboring *FKS* mutations isolated from plates containing the first micafungin and anidulafungin concentrations considered as resistant by EUCAST. Nevertheless, a significantly higher *FKS* mutant rate was found after micafungin exposure than after anidulafungin selection.

*In vitro* micafungin and anidulafungin exposure allowed the selection of different *FKS* mutations grown at different concentrations and even at the same one for some strains. S663P was the most frequently found mutation following exposure to both echinocandins, although in previous *in vitro* selection studies ΔF659 was the most prevalent one (Bordallo-Cardona et al., 2018c; Shields et al., 2019), which was the second most common one in our study.

We found 53 colonies with MICs onefold or twofold above the established breakpoint for anidulafungin that did not carry any *FKS* mutations. This finding was especially detected after exposure to anidulafungin, as 50 colonies out of 258 isolated were anidulafungin resistant but *FKS* wild-type, while only three out of 296 colonies analyzed after micafungin exposure had this categorization. This similar case and also echinocandin susceptible isolates harboring *FKS* substitutions have been previously found, both in clinical isolates and after *in vitro* exposure (Castanheira et al., 2014; Pham et al., 2014; Shields et al., 2015, 2019; Locke et al., 2016). Precisely, anidulafungin resistance, according to EUCAST breakpoints, was vastly sensitive (100%; all colonies that were *FKS* mutant showed a MIC value above its clinical breakpoint for EUCAST) but showed lower specificity (80.6%; as 53 colonies out of a total of 273 that were *FKS* wild-type showed MICs above the EUCAST breakpoint) than in another study (Shields et al., 2019) for the identification of *in vitro* selected *FKS* mutant colonies. Micafungin showed higher sensitivity (99.3%; 279 out of 281 isolates that harbored *FKS* mutations were micafungin resistant according to EUCAST breakpoint) and similar specificity (99.8%; only one *FKS* wild-type colony was micafungin resistant) to those reported in that study. Taken together, this confirmed that both antifungals are suitable echinocandin resistance markers for *C. glabrata*, unlike caspofungin (Shields et al., 2013; Eschenauer et al., 2014).

We concluded that the development of echinocandin resistance in *C. glabrata* after *in vitro* exposure to micafungin and anidulafungin has no association with specific genotypes. Results obtained in all these *in vitro* studies on how echinocandin susceptible *C. glabrata* strains are able to develop resistance after exposure to low echinocandin concentrations supports the fact that *C. glabrata* is able to colonize and survive in certain reservoirs of the human body, such as the abdomen (Shields et al., 2014), the peritoneum (Grau et al., 2015), the gastrointestinal tract (Healey et al., 2017) or the mucosal surfaces (Jensen et al., 2015), due to long-term penetration of echinocandins in lower concentrations than those that prevent resistance acquisition. Since sometimes this required dose could lead to toxicity, the use of newly developed drugs that target the 1–3-β-D-glucan synthase, such as ibrexafungerp (Scynexis, Jersey City, NJ, United States), which has shown potential effectiveness against echinocandin resistant *C. glabrata* isolates (Wiederhold et al., 2018), or rezafungin (Cidara, San Diego, CA, United States), which has an extended-interval administration due to its improved pharmacodynamics (Sandison et al., 2017) could help to overcome echinocandin resistance. Nevertheless, *C. glabrata* strains have been *in vitro* exposed to both drugs, leading to similar results than those against other echinocandins (Locke et al., 2016; Jimenez-Ortigosa et al., 2017), so further research for new compounds that have a role on novel mechanisms of action is assured.

In summary, the present study analyzes the relevance of certain hypothesis raised on the increase of echinocandin resistance in *C. glabrata*, and sheds light on several important aspects related to its acquisition and development, both in genetically related serial isolates from the same patient and after *in vitro* exposure to micafungin and anidulafungin.

## Data Availability

All datasets generated for this study are included in the manuscript and/or the supplementary files.

## Author Contributions

AA-I and OR-M conceived and designed the experiments. OR-M, PN-R, LB-M, GM-C, and LL-P performed the experimental work. OR-M and AA-I analyzed the data. IS-R and AP-A provided clinical isolates. OZ provided materials. OR-M and AA-I wrote the manuscript. All authors read, edited, and approved the final version of the manuscript.

## Conflict of Interest Statement

In the past five years, AA-I has received grants from GILEAD, F2G, and Scynexis and personal fees for educational conferences from Pfizer Astellas, MSD, and GILEAD outside the submitted work. The remaining authors declare that the research was conducted in the absence of any commercial or financial relationships that could be construed as a potential conflict of interest.

## Footnotes

1 http://www.eucast.org/fileadmin/src/media/PDFs/EUCAST_files/AFST/Files/EUCAST_E_Def_7_3_1_Yeast_testing__definitive.pdf (accessed April 8, 2019).

2 http://www.eucast.org/astoffungi/clinicalbreakpointsforantifungals/

3 http://www.ncbi.nlm.nih.gov/

4 https://pubmlst.org/cglabrata/
